# Towards an East Asian model of climate change awareness: A questionnaire study among university students in Taiwan

**DOI:** 10.1371/journal.pone.0206298

**Published:** 2018-10-25

**Authors:** Bruno Di Giusto, Joseph P. Lavallee, Tai-Yi Yu

**Affiliations:** 1 Journalism and Mass Communication Program, Ming Chuan University International College, Taipei, Taiwan; 2 International Business and Trade Program, Ming Chuan University International College, Taipei, Taiwan; 3 Department of Risk Management and Insurance, Ming Chuan University, Taipei, Taiwan; Indiana University, UNITED STATES

## Abstract

East Asia emits more greenhouse gases into the atmosphere than any other region, yet little is known about attitudes towards climate change in this region. A cross-sectional survey investigating climate change knowledge, concern and behavior change was administered to 1118 university students at nine universities across Taiwan in June 2016. Knowledge was assessed with a 15-item quiz while concern and behavioral change were self-reported on 5-point Likert scales. The relationship of these three variables with various socio-demographic variables was investigated through Kruskal-Wallis tests and ordinal logistic regressions. Knowledge was homogeneous by region but differed sharply by socioeconomic position. Concern appears high by international standards, with 65% reporting being “somewhat concerned” and 28% being “very concerned,” while climate change denial was negligible. Students expressing greater concern were more likely to be from eastern and southern Taiwan, regions more vulnerable to extreme weather events. However, these high concern levels did not translate into action, as only 38% of respondents reported “some” and 11% reported “very much” behavioral change in response to climate change. Higher levels of behavioral change were reported by students expressing greater concern and students with *lower* levels of climate change knowledge. In contrast with studies of Western societies, our findings suggest an East Asian model in which the conflict between economic growth and the environment is playing out in different ways, such that the crucial need is for policy leadership and not more education.

## Introduction

The potential threats posed by climate change in the coming decades are eliciting growing global awareness and concern. Rising sea levels and changes in precipitation and temperature patterns are creating more favorable conditions for diseases and threatening food security [1–4]. If the frequency of conflicts increases with the warming of the planet, these risks may be amplified by weakened governance capacity and economic disruptions [5–6]. For the 21^st^ century, climate change appears set to be a defining international public crisis.

Responding to these potential dangers will require massive, sustained mitigation efforts to reduce growth in greenhouse gas levels, and adaptation efforts to respond to the impacts. Public support will be essential for the changes required. In recent decades, researchers have tracked trends in public knowledge about and perception of climate change. However, this literature has disproportionately focused on Western countries, particularly the United States. Far less research has appeared on East Asia, a region currently responsible for nearly 35% of global CO_2_ emissions–a higher percentage than the US and the EU combined (Table 1; in this article, East Asia refers to China, Japan, South Korea and Taiwan). East Asian countries all have large, export-oriented industrial sectors and high levels of per capita emissions (Table 1). Further, these economies have failed to significantly reduce emissions, with climate policy for all of them being graded “poor” or “very poor” in the Germanwatch 2018 climate change performance index [7]. China, at a lower level of development, is already the world’s largest greenhouse gas emitter. While much of this is due to production to meet Western consumption demand, a shift towards domestic consumption in China [8] implies that East Asia will need to reduce emissions related to both production and consumption. What role will public perceptions related to climate change play in the success or failure of this transition? International comparative studies of public attitudes indicate that generalizations about East Asia cannot be made from studies of Western countries [9]. East Asian survey respondents, compared to those in the US, appear to place greater value on the environment and show higher acceptance of climate change science [10–13]. However, the number of studies is limited and has not yet extended to the question of willingness to change.

**Table 1:** Economic and environmental indicators: East Asia vs. other global regions^a^.

	% Share of Global GDP	% Share of Global Population	% Share of Global Greenhouse Gas Emissions^b^	Greenhouse Gas Emissions per capita Tons CO_2_	CCPI 2017
**East Asia**^c^	**21.73%**	**21.69%**	**34.41%**	**7.08**	**56**^d^
China/HK	16.98%	18.92%	28.21%	6.66	48
Japan	4.38%	1.75%	3.67%	9.35	60
South Korea	1.67%	0.70%	1.75%	11.26	61
Taiwan	0.66%	0.32%	0.77%	10.68	52
**United States**	**15.94%**	**4.40%**	**15.99%**	**16.22**	**43**
**European Union**	**23.90%**	**7%**	**9.76%**	**6.22**	**20**^e^
**Rest of World**	**38.43%**	**66.91%**	**39.84%**		
World	72,907.58 Bn USD	7248.66 Million	32,381.04 Mt of CO2	4.47	

^a^ Data for columns 1–4 was drawn from International Energy Association 2014 [14]; data for the fifth column was drawn from the CC Performance Index 2017 [15].

^b^ Energy-related emissions

^c^ Excluding North Korea and Mongolia, two East Asian countries with low emissions.

^d^ Calculated by averaging the scores of the 4 countries and looking at the ranking of the calculated new score CC Performance Index (CCPI) 2017 [15].

^e^ Calculated by averaging the scores of the 28 countries and looking at the ranking of the calculated new score CC Performance Index 2017 [15].

With continued economic development, our best proxies for understanding the likely trajectory of emissions in China come from other East Asian countries. Taiwan, closest culturally to China, is a case of special interest. With its energy-intensive industrial base, Taiwan is responsible for nearly 1% of global emissions and has one of the highest levels of per capita emissions in the region [16, 17]. Taiwan has set a target of reducing emissions by 50% of the 2005 level by 2050. However, Taiwan’s reliance on industry and its decision to close its remaining nuclear reactors (generating 18% of Taiwan’s electricity as of 2016) by 2025, means that meeting this goal will require significant increases in energy efficiency and reduced consumption [18]. Research in the US and Europe suggests that reductions of nearly 40% through energy saving behaviors are possible [19]. If policymakers seek to aggressively curtail consumption-related emissions, will the public support such policies?

The present study seeks to help answer such questions by summarizing key findings from a broader investigation of attitudes towards climate change in Taiwan. Recent studies in Taiwan suggest that a high degree of concern about climate change exists in the adult population [20] but that this concern has not translated into high levels of behavioral change [21]. Taiwan’s policymakers have increasingly turned to education to promote sustainable development, culminating in the Environmental Education Act in 2011 [22], and Taiwan’s public-school teachers have been shown to have adequate levels of environmental literacy [23]. Are these efforts bearing fruit in terms of student risk perception? In this paper, we investigate the relationships between climate change literacy, concern and behavioral change among university students. The longer time horizon of students makes them more vulnerable to the effects of climate change, and, from a threat-reduction perspective, students form a critical target population as they are at a stage at which lifelong habits related to mitigation and adaptation are being formed. In Taiwan, this population is particularly relevant, as about 71% of people in this age group attend tertiary education [24]. As the next generation of citizens and consumers, a better understanding of their attitudes will also help in forecasting future trends. Surprisingly few studies have attempted to assess the knowledge and attitudes of university students towards climate change [25] and none have attempted to sample across all majors and degrees, much less across a country. Further, many used small samples (under 400) and/or were designed to respond to challenges faced in Western countries, particularly the need for education to overcome climate change denialism [26–28]. Ours is the first study to cover knowledge, concern and behavioral change in a single investigation and to include students from across all majors and degrees, across all years of university study, across universities ranked at different levels and across all geographical regions. In this study, we seek to identify the relationships between climate change knowledge, concern and behavioral change, as well as to determine whether social background factors influence these variables.

## Methods

### Participants

A total of 1118 participants (mean age = 21.4, SD = 2.4) completed the questionnaire. Participants were selected based on a sampling design considering student hometown region, curriculum (student major) and university ranking (Table 2). The study was approved by the local institutional review board (Research Ethics Committee of National Taiwan University, NTU-REC No. 201506ES037) and carried out in accordance with the relevant guidelines and regulations. Participants gave their free and informed written consent prior to completing the survey.

**Table 2:** Demographic characteristics of sample vs. student population in Taiwan.

	Sample^a^	Taiwan–Tertiary Education, 2015
	(n = 1118)	N = 1,332,445 [29]
**Gender** (NAs = 59)		
Male (n = 448)	42.3%	M = 49.6% [29]
Female (n = 611)	57.7%	F = 50.4%
**Household Income** (NA = 531)		
Below NT$30,000 (n = 72)	12.3%	5^th^ Q 26,693 [30]
NT$30,001—NT$55,000 (n = 162)	27.6%	4^th^ Q 48,980
NT$55,001—NT$80,000 (n = 152)	25.9%	3^rd^ Q 69,737
NT$80,001 and above (n = 201)	34.2%	2^nd^ Q 94,986
		1^st^ Q 161,643
**Student Hometown Region** (NA = 165)		
Centre (n = 176)	18.5%	24.1% [31]
East (n = 26)	2.7%	2.2%
North (n = 557)	58.4%	46.3%
South (n = 194)	20.4%	27.1%
**Mother’s Education** (Highest level completed) (NA = 154)		
Middle School or Below (n = 122)	12.7%	3.6% [32]
High School (n = 406)	42.1%	34.9%
Associate Degree (n = 185)	19.2%	13.6%
BA or Higher (n = 251)	26.0%	17.9%
**Student Education** (NA = 72)		
Freshmen (n = 228)	21.8%	21.7% [33]
Sophomore (n = 241)	23.0%	20.9%
Junior (n = 288)	27.5%	20.2%
Senior (n = 200)	19.1%	19.8%
Master & PhD (n = 89)	8.5%	17.5%
**Student Major Area** (NA = 157)		
Humanities (n = 256)	26.6%	18.7% [34]
Social Sciences (n = 357)	37.1%	38.5%
Sciences & Technology (n = 318)	33.1%	42.8%
Cosmetic & Beauty Science (n = 30)^b^	3.1%	Not available
**Student Political Party** (NA = 582)		
Pan-Blue (KMT, PFP) (n = 118)	22.0%	
Pan-Green (DPP, NPP) (n = 155)	28.9%	Not available
Environmental Parties (GRN, TRP, GSD) (n = 8)	1.5%	
Others (CCP, NPS, PAN, WFP, OTH) (n = 65)	12.1%	
Non-affiliated (n = 190)	35.4%	

^a^ Percentages for sample statistics represent percentages of valid responses, after excluding non-responses or other non-valid responses (marked “NA” in column on left).

^b^ Students in Cosmetic & Beauty Science were removed from the Sciences & Technology category after a review of their curricular content.

### Measures

#### Dependent variables

Climate-change related knowledge, risk perception and willingness to change behavior among university students in Taiwan was assessed through a three-part Chinese-language (traditional script) questionnaire in June 2016. We conducted a pilot test with 231 participants along with a follow-up series of focus-groups to investigate item quality. The results were used to select the final set of questions included in the questionnaire. Knowledge was measured with fifteen multiple-choice questions covering basic scientific and social aspects of climate change (Cronbach’s alpha internal consistency reliability was marginally acceptable at 0.645; all items are provided in Chinese and English in Supporting Information S1 Text). We present here the percentage of correct answers. To measure risk perception, we asked fourteen questions. In this paper, we present results to the question “How concerned are you, if at all, that global climate change will harm you personally at some point in your lifetime?” given the response options “Very concerned,” “Somewhat concerned,” “Not too concerned,” “Not at all concerned,” “Climate change does not exist,” and “Don’t know.” To measure readiness to make behavioral changes, we asked four questions. In this paper, we present results to the question “How much have you changed your behavior because of climate change—not at all, a little, some or very much?”

#### Explanatory variables

Participants were asked to report their gender, household income in New Taiwan Dollars (NTD), hometown region, mother and father’s level of education, student (personal) education, student major area, student political party; university ranking was also included as a variable (S1 Table). Mother’s level of education was used as it displayed a stronger relationship to the outcome variables than did father’s level of education. We collapsed student majors into the three broad categories used by Taiwan’s Ministry of Education: Humanities, Social Sciences, and Science and Technology. However, we subsequently created a fourth category for “Cosmetic and Beauty Science,” (originally included in the Science and Technology category) following a review of the curricular content of this major. For political affiliation, students were asked to indicate the political party they most identified with. In Taiwan, political parties are often discussed in terms of the ‘pan-Blue’ and ‘pan-Green’ camps, divided primarily by their attitudes toward relations with China. The parties of the pan-Blue camp—the Kuomintang (KMT) and the People’s First Party (PFP)—tend to prefer close Taiwan-China relations with re-unification as an eventual goal while the pan-Green parties—the Democratic Progressive Party (DPP) and New Power Party (NPP) tend to lean toward pro-independence policies. Note that ‘Green’ here does not have a pro-environment connotation. We refer to Taiwan’s small environment-centered parties as simply ‘Environmental Parties.’ The categories used, and the results are provided in Table 2, which also indicates differences between our sample and the population of Taiwanese tertiary students for the categories reported.

### Data analysis strategy

We investigated three dependent variables in the study: (1) climate change knowledge; (2) climate change concern; and (3) behavioral change related to climate change. As climate change knowledge scores were non-normally distributed, and as the other dependent variables analyzed were ordinal, the knowledge scores were transformed into ordinal categories for greater coherence in the treatment of our outcome variables. We first conducted Kruskal-Wallis H tests to assess the effect of each individual explanatory variable on the dependent variables. We then developed ordinal logistic regression models to estimate the effect size of the explanatory variables within integrated models. The best fitting model for each dependent variable was chosen using goodness of fit statistics. All analyses were performed with the SPSS statistical package [35]. A more detailed description of the statistical analyses performed is provided in S2 Text.

## Results

### Knowledge about climate change

On the knowledge quiz, the average percent correct score was 62.7%. Scores differed significantly based on gender (men scored significantly higher), household income (students with higher incomes scored significantly higher), mother’s level of education (students whose mothers had higher levels of education scored significantly higher), student education (students who had completed more years at university scored significantly higher), student major area (science and technology students scored significantly higher than humanities students, who outscored social science students, with cosmetics and beauty science students having the lowest scores), student political party (non-affiliated students scored significantly higher than pan-Green), and university ranking (students at higher ranked universities scored significantly higher). Similar results were found when knowledge was treated as an ordinal variable, with the difference that student hometown region also became significant (students from the north scoring significantly higher than students from the eastern part of the island). Full results appear in Tables A and B in S2 Table.

In the ordinal logistic regression model for climate change knowledge, socioeconomic position was critical as only university ranking and family income significantly predicted knowledge scores. Students from first-tier universities had the highest climate change knowledge scores; being in a second-tier or third-tier university was associated with a decrease in the odds of having a high level of climate change knowledge. Further, students whose household income was inferior to 30,000 NTD had decreased odds of having a high level of climate change knowledge relative to students whose household income was greater than 80,000 NTD. Odds ratios are given in Table 3.

**Table 3:** Ordinal regression on climate change knowledge.

	Odds Ratios^1^ for Knowledge (95% Confidence Interval)
**University Ranking**^2^ (First Tier as Reference)	
Second Tier	0.168 *** (0.111 to 0.253)
Third Tier	0.058 *** (0.036 to 0.094)
**Family Income** (Above 80,000 as Reference)	
< 30,000 Monthly	0.479 ** (0.282 to 0.814)
30,000 to 50,000 Monthly	0.864 (0.580 to 1.286)
50,000 to 80,000 Monthly	0.807 (0.539 to 1.207)

*p < .05,

**p < .01,

***p < .001.

^1^Odds ratios indicate the odds of a dependent variable outcome given a particular value of the independent variable compared to the odds of the same outcome occurring for the value of the independent variable represented by the reference group.

^2^University ranking and family income, both ordinal variables, were treated as categorical in a second regression to generate the odds ratios in this table. When this was done, family income no longer had a significant effect in the model ([χ^2^(3) = 7.566, *p* = .056]).

### Concern/Risk perception

In terms of concern, 1% were “not at all concerned”, 6% were “not too concerned”, 65% were “somewhat concerned” and 28% were very concerned (Table C in S2 Table). Of 1118 students, only four chose “Climate change does not exist.” Level of concern differed significantly based on student hometown region (students in the north were significantly less concerned than students from the south), major area (science and technology students were significantly less concerned than social science students) and political party (students affiliated with other parties and non-affiliated students were significantly less concerned than students affiliated with environmental parties) (Table C in S2 Table).

Through ordinal logistic regression, we determined the effects of the same set of variables *and* climate change knowledge on the likelihood that students would report being more concerned. Hometown region, political party and student major all significantly predicted degree of concern. Students from eastern and southern Taiwan, and students majoring in the social sciences were more likely to report higher levels of concern (Table 4). In terms of political affiliation, students reporting “other” political parties were the *least* likely to report high levels of concern.

**Table 4:** Ordinal regression on degree of concern.

	Odds Ratios^1^ for Concern (95% Confidence Interval)
**Region** (South as Reference)	
Central	.353*** (.187 to .667)
East	.971 (.285 to 3.304)
North	.493** (.303 to .802)
**Political Party** (None as Reference)	
Pan-Green	0.985 (.597 to 1.626)
Environmental	5.064 (.858 to 29.891
Pan-Blue	1.249 (.741 to 2.105)
Others	.430* (.220 to .838)
**Major** (Science & Technology as Reference)	
Others	.722 (.206 to 2.531)
Humanities	1.080 (.637 to 1.832)
Social Sciences	1.939** (1.209 to 3.110)

*p < .05

**p < .01

***p < .001.

^1^ Odds ratios indicate the odds of a dependent variable outcome given a particular value of the independent variable compared to the odds of the same outcome occurring for the value of the independent variable represented by the reference group.

### Behavioral change related to climate change

In response to the question “How much have you changed your behavior because of climate change?”, 3% responded “not at all”, 43% responded “a little”, 38% responded “some” and 11% responded “very much” (with approximately 5% not responding) (Table D in S2 Table). The degree of behavioral change differed significantly based on household income (however, post hoc Dunn pairwise comparisons failed to identify significant differences between income categories), student major area (social science students reported significantly higher levels of behavioral change than both humanities and science and technology students) and university ranking (students from low and middle tier universities reported significantly higher levels of behavioral change than did students at the highest tier universities). Detailed results are provided in Table D in S2 Table.

Through ordinal logistic regression, we determined the effects of the same set of demographic variables plus climate change knowledge and concern on the likelihood that students would report higher levels of behavioral change in response to climate change. Knowledge and concern emerged as the key predictors. Surprisingly, knowledge showed a slight negative relationship with behavioral change: students with lower scores reported more behavioral change while students with higher scores reported less behavioral change. Concern, as expected, showed a strong positive relationship with reported level of behavioral change. The best fitting model included both hometown region and student major. However, these two variables were only marginally significant. Students from the south reported the highest levels of change; students from eastern Taiwan were significantly less likely to report change than students from the south. Odds ratios appear in Table 5.

**Table 5:** Ordinal regression on degree of behavioral change.

		Odds Ratio^1^ for Behavioral Change (95% Confidence Interval)
**Knowledge** (percent correct)		.987** (0.978 to 0.995)
**Concern**		3.234*** (2.505 to 4.176)
**Major**	Others	2.123 (.952 to 4.735)
Reference Group: Sci Tech	Humanities	.813 (.570 to 1.159)
	Soc Sci	1.237 (.886 to 1.727)
**Region**	Center	0.890 (0.584 to 1.355)
Reference Group: South	East	.332* (.136 to .815)
	North	.763 (.542 to 1.073)

*p < .05

**p < .01

***p < .001.

^1^ Odds ratios indicate the odds of a dependent variable outcome given a particular value of the independent variable compared to the odds of the same outcome occurring for the value of the independent variable represented by the reference group.

## Discussion

This paper investigated the relationships between climate change knowledge, the degree of concern and reported behavioral change in response to climate change among 1118 university students in Taiwan. The level of concern towards climate change is high amongst Taiwanese students but only moderate levels of behavioral change were reported. In terms of demographics, students from higher ranked universities scored significantly higher on the knowledge quiz, while students from households in the lowest income category scored significantly lower. However, knowledge was not a predictor of concern and was actually a *negative* predictor of behavioral change. The degree of concern was mainly predicted by student hometown region (students from the south and east reported being more concerned) and by student major (social science students reported being more concerned). The level of concern, in turn, was the strongest predictor of actual behavioral change. These results suggest that lack of knowledge is not an obstacle to behavioral change. Further, although greater concern predicts higher levels of behavioral change, the high degree of concern found in Taiwan has not, as of yet, translated into correspondingly high levels of behavioral change, a result consistent with an earlier study of Taiwan’s adult population [21]. Optimistically, the lack of behavioral change to date might be remedied by more active policy leadership. The university years, especially in Taiwan where a high percentage of students attend university, may represent an ideal opportunity.

A key finding of the paper is the unexpected failure to find a positive relationship between knowledge and either concern or behavioral change. Indeed, there was actually a slight *negative* relationship between knowledge and reported behavioral change. Research across a number of societies has shown a positive relationship between education and both environmental concern and disaster preparedness [36]. In the US, research has found an interaction between political beliefs, with a positive relationship between climate change knowledge and concern among Democrats but a negative relationship among Republicans [37, 38]. In an earlier risk perception survey of the entire adult population of Taiwan, education was generally found to predict concern towards climate change [20].

Based on this literature, we were surprised to find no relationship between knowledge and concern and a negative relationship between knowledge and behavioral change in the present study. One possibility is that a positive relationship does exist across an entire society or when comparing different societies, where knowledge levels vary widely. In our sample of university students in Taiwan, where climate change education is part of the curriculum, a basic level of knowledge existed across the sample as indicated by the absence of differences by region. Given this basic level, further increases in knowledge may no longer predict increases in concern or behavioral change. In Taiwan, 70.9% of the relevant population moved on to tertiary education in 2015 [24]. It is possible that if the remaining 29.1% had been included in the study, a positive relation between knowledge and concern would have been found.

We propose two hypotheses to explain the *negative* knowledge-behavior change relationship found in our study. First, it may have resulted from the *positive* relationship between family income and knowledge combined with the *negative* relationship between family income and behavioral change (Tables A and D in S2 Table). The latter relationship may be due to a feeling of greater vulnerability to extreme weather events among lower income students or it may be that lower income students view the behavioral changes reported in this study as money-saving. Second, there might be a threshold at which more education counter-productively increases feelings of helplessness by increasing the understanding that responses to climate change require society-wide programs, rather than changes to individual behavior. Further research could usefully explore the relationship between knowledge and concern/behavioral change across the entire range of knowledge levels.

Turning to concern, a further significant finding of our study is that a strong positive relationship did appear between concern and behavioral change. In Taiwan, concern is high, and it correlates with behavioral change. This must be regarded as an achievement of Taiwan’s educational program around climate change [22, 23]. Nonetheless, some significant differences in concern were found based on our demographic variables. First, students expressing higher concern levels were more likely to come from southern and eastern Taiwan, regions with higher levels of exposure to weather-related hazards (although no major extreme weather events were recorded in Taiwan during the period of our study). Storms typically originate to the southeast of Taiwan, between the Caroline Islands and the Philippines, with an average three to four storms occurring between July and October. The presence of a mountain range running from the north to south serves as a natural barrier lessening the impact of these storms on the western part of the island. Among Taiwan’s adult population, Sun and Han found that direct experience of weather-related disasters was not associated with climate change risk perception. However, more general typhoon-related anxiety was related to perceived risks towards climate change [20], possibly explaining the higher levels of concern shown by our students from the south and east. This is consistent with past research linking vulnerability and concern [39] though others have failed to find a connection [40]. Future research might help clarify the nature of this relationship in Taiwan.

A second difference is that social science students were significantly more likely to report higher levels of concern. We suspect that this reflects curricular differences, with students in the social sciences exposed to more discussions of the impacts of climate change on society. (As students often select majors based on college entrance exam scores in Taiwan, we do not believe that our results reflect pre-existing personal attitudes, as they might in western countries.) A third and final difference is the lack of a finding: concern was not related to support for any of Taiwan’s main political parties. This is consistent with Sun and Han’s findings of generally weak associations with political preference and no clear differences in risk perception based on support for the main political groupings [20]. In short, attitudes towards climate change do not appear to be based on politics in Taiwan.

Our results support recent studies contrasting beliefs and attitudes in East Asia and the West. In two recent large-scale university-level surveys in China and the US [11,12], Chinese students were more likely than their US counterparts to believe that climate change is happening and caused by human activities. Chinese students expressed no clear tendency towards ‘climate change denialism’, demonstrated a more accurate understanding of the science and expressed greater concern than US students [for further results on US students, see 25–27]. In our study in Taiwan, climate change denial was almost non-existent (only four of 1118 respondents). Jamelske et al. [12] offer two systemic explanations for US-China differences: state control in China preventing the emergence of denialism and Chinese traditional respect for educational authorities and science. Although in democratic Taiwan there is no similar degree of state control over public discourse, Taiwan may share with China a respect for science and educational authorities.

Furthermore, these trends among students appear to also be true for the broader adult populations of China [10, 41] and Taiwan [20, 21]. Taken together, these findings suggest the need for a different understanding of the role of public awareness and climate change policy in this critical part of the world. The generally high levels of concern and the lack of climate change denialism in East Asia, combined with the insufficient policy response to climate change (Table 1), suggests that public opinion, and thus individual psychology, may play less of a role in national policies in this region than has been suggested for Western countries [42–44]. East Asians demonstrate higher levels of awareness, yet the region continues to be the world’s largest emitter of greenhouse gases and fares poorly in international comparisons of responses to climate change [7].

Chou has recently framed Taiwan’s climate policies in terms of its status as a newly-industrialized country, prioritizing economic growth and insulating the policy-making process from public opinion [21]. Similarly, Shih discusses opposition to the passage of the Greenhouse Gas Reduction Act in terms of the opposition of energy-intensive industrial firms, established on the island from years of a pro-development policy of low-energy prices, in combination with government ministries focused on economic ‘competitiveness’ and a public that views climate change as a real but distant threat [45].

We further suggest that Taiwan is one instance of a broader East Asian model, the key feature of which is the commanding role of the “developmental state” [46], which contrasts sharply with the “regulatory state” in countries like the US and the UK. In the US, the conflict between economic interests and the environment has played out in the political sphere. Vested economic interests funded efforts to generate doubt about climate change and thereby hinder or prevent policy responses [47]. As a result, educators, left on the defensive and forced to focus on techniques to convey accurate information, are still struggling to raise awareness and concern prior to instilling environmentally friendly habits. In East Asia, by contrast, public opinion is often disregarded and the conflict between vested economic interests and the environment is played out not in the political sphere but rather as inter-ministerial disputes *within* governments. With the exception of China’s coal exports, the East Asian economies are also oil, LNG and coal importers, with no significant fossil fuel producing interests. For these reasons, active private-sector disinformation campaigns are not a factor. This explains the finding, paradoxical from a western perspective, of populations knowledgeable and concerned about climate change but economies still locked into high levels of emissions. The resulting discrepancy between government-sponsored education and recommendations (made by ministries of education and the environment) and actual government policies on the environment (shaped by ministries responsible for economic policy) may also lead to a lack of trust in government [21] which could partially explain the lack of behavioral change found in this study. It raises the further point that opposition to environmentally damaging policies will necessarily take a different form in East Asia, as discussed and illustrated in Chou’s recent discussion of the 2010–11 movement to stop the construction of a naphtha cracking plant in Taiwan [48]. Given the central importance of East Asia to the problem of climate change, we feel that future research should continue to focus on the specific dynamics of policy formation and the role of public attitudes in the region, without assuming similarity to western norms.

### Limitations

This study did not use a representative sample. Table 1 compares the characteristics of our sample with those of the student population in Taiwan. Interpretations of our results should thus be made with appropriate caution. Also, our study was based on self-reported levels of concern and changes in behavior and these responses are subject to biases (e.g., social desirability bias). Finally, as our data was cross-sectional, we cannot make strong causal claims concerning the correlations found here, although we hope that our regression analyses have shed light on the possible relations underlying our findings.

## Conclusions

Mitigating the potential hazards posed by climate change will require significant behavioral changes among the public. Programs encouraging such changes will need to be “tailored to the unique context of each country” as the level of public knowledge and perception within each country has its own “relatively unique set of correlates” [49]. This study supports the existence of a distinct East Asian model of public opinion in which knowledge and concern exist at high levels and in which climate change denialism is not relevant. The need for greater education often emphasized in studies of Western countries [25–27] seems less urgent in Taiwan and any further education should focus on concrete adaptation and mitigation strategies and solutions. Optimistically, the high levels of knowledge and concern in East Asia bodes well for future support of policies responding to climate change. What is needed in the East Asian context, then, is policy leadership which translates existing levels of knowledge and concern into behavioral change.

## Supporting information

S1 Table Relative ranking of the universities in the sample. (DOCX)

S2 Table Kruskal-Wallis test results. (DOCX)

S1 Text Climate Change Knowledge Quiz (Chinese and English). (DOCX)

S2 Text Details of the statistical analysis. (DOCX)
