# Supplementary material for: Towards an East Asian model of climate change awareness: A questionnaire study among university students in Taiwan
Source: PLoS One. 2018 Oct 25;13(10):e0206298. doi: 10.1371/journal.pone.0206298 (PMC6201920; doi:10.1371/journal.pone.0206298)
Supplement: S1 Table — (DOCX) [file pone.0206298.s001.docx]

**S1 Table. Relative ranking of the universities in the sample**

| University | Ranking* | Number of Survey Participants  (1118) |
| --- | --- | --- |
| **1** | High | 105 |
| **2** | High | 135 |
| **3** | High | 140 |
| **4** | Middle | 73 |
| **5** | Middle | 253 |
| **6** | Middle | 114 |
| **7** | Lower | 123 |
| **8** | Lower | 78 |
| **9** | Lower | 97 |

*****Universities ranked amongst the top 20 universities in Taiwan were classified as “High”; those ranked between 21 and 50 were classified as “Middle” and those ranked below 50 were classified as “Lower”. Classifications were found to agree across two separate ranking systems.^1,2^

**References**

1. 2015-16 Ranking by Country: Taiwan. University Ranking by Academic Performance website. <http://www.urapcenter.org/2015/country.php?ccode=TW&rank=all> Cited on November 27, 2017.

2. Now News Website. 2016 University Rankings. <https://www.nownews.com/news/20160929/2254216> Cited on November 27, 2017.
