# Supplementary material for: Towards an East Asian model of climate change awareness: A questionnaire study among university students in Taiwan
Source: PLoS One. 2018 Oct 25;13(10):e0206298. doi: 10.1371/journal.pone.0206298 (PMC6201920; doi:10.1371/journal.pone.0206298)
Supplement: S2 Table — (DOCX) [file pone.0206298.s002.docx]

**S2 Table A. Climate change knowledge: Percentage of items correct out of 15, by demographics^*^**

|  | | | |
| --- | --- | --- | --- |
|  | **Categories** | **Percent Correct**  **(Mean ± Standard Deviation)** | ***p*-value of**  **Kruskal Wallis**  𝟀^2^ **test** |
| All |  | 62.7 ± 18.1 |  |
| Gender | Male  Female | 64.8 ± 18.1  61.3 ± 17.5 | **<0.0006** |
| Household Income | < NT$30,000  NT$30,001 - NT$55,000  NT$55,001 - NT$80,000  ≥ NT$80,001 | 51.8 ± 19.9^a^  60.1 ± 17.8^b^  63.7 ± 16.4^bc^  65.3 ± 18.0^c^ | **5.43 x 10^-07^** |
| Student Hometown Region | Centre  East  North  South | 62.1 ± 17.9  58.5 ± 24.4  62.4 ± 17.9  66.0 ± 17.0 | 0.10 |
| Mother’s Education  (Highest *completed*) | Middle School or Below  High School  Associate’s (Jr. College)  BA or Higher | 57.9 ± 17.3^a^  62.0 ± 17.9^ab^  65.4 ± 16.9^bc^  66.1 ± 17.3^c^ | **4.28 x 10^-05^** |
| Student’s Current Educational Level | Freshmen  Sophomore  Junior  Senior  Master & PhD | 59.5 ± 21.3^a^  62.0 ± 16.8^a^  63.3 ± 16.6^ab^  64.0 ± 17.7 ^ab^  69.1 ± 12.6^b^ | **<0.0004** |
| Student Major Area^a^ | Humanities  Soc Sci  Sciences & Tech  Cosmetics & Beauty Sci | 64.0 ± 15.6^c^  59.9 ± 17.0^b^  69.7 ± 15.7^d^  41.3 ± 14.5^a^ | **2.2 x 10^-16^** |
| Student Political Party | Pan-Blue (KMT & PFP)  Pan-Green (DPP & NPP)  Environmental parties  Others  Non-affiliated | 63.3 ± 18.4^ab^  58.5 ± 19.8^a^  76.7 ± 17.5^ab^  59.1 ± 18.8^ab^  66.0 ± 16.1^b^ | **0.001** |
| Univ. Ranking | High  Mid  Low | 75.3 ± 13.2^c^  60.4 ± 14.9^b^  50.0 ± 17.6^a^ | **< 2.2 x 10^-16^** |

Note: Boldface indicates statistical significance (*p* < 0.05). The different superscript letters in the Percent Correct column indicate statistically significant difference between pairs determined by post hoc Dunn tests.

^*^Knowledge scores for students who did not respond for particular demographic variables were tested against scores for responding students; no significant differences were found.

**S2 Table B. Climate change knowledge (ordinal), by demographics**

|  | | | | | | |
| --- | --- | --- | --- | --- | --- | --- |
|  |  | **Climate Change Knowledge Scores** | | | |  |
|  | **Categories** | **0 – 40%**  **Correct**  **(%)** | **41 – 60%**  **Correct**  **(%)** | **61 – 80%**  **Correct**  **(%)** | **81 – 100%**  **Correct**  **(%)** | ***p*-value of**  **Kruskal Wallis** 𝟀^2^ **test** |
| All |  |  |  |  |  |  |
| Gender | Female  Male | 20.3  12.9 | 27.1  29.2 | 35.6  43.8 | 16.9  14.1 | **.002** |
| Household Income | < NT$30,000^a^  NT$30,001 - 55,000^b^  NT$55,001 - 80,000^b^  ≥ $80,001^b^ | 31.9  14.2  13.2  11.9 | 41.7  42.6  30.9  29.9 | 22.2  34.0  47.4  45.3 | 4.2  9.3  8.6  12.9 | **< .001** |
| Student Hometown Region | Center^ab^  East^a^  North^b^  South^ab^ | 14.2  30.8  14.7  8.8 | 37.5  7.7  33.4  29.9 | 37.5  57.7  42.2  46.4 | 10.8  3.8  9.7  14.9 | **.024** |
| Mother’s Education  (Highest *completed*) | Up to Middle School ^a^  High School ^ab^  JR College ^b^  BA or Above ^b^ | 18.0  15.3  9.7  11.2 | 38.5  33.5  29.2  30.7 | 37.7  42.4  49.2  42.6 | 5.7  8.9  11.9  15.5 | **.001** |
| Student’s Current Educational Level | Freshmen ^ac^  Sophomore ^ab^  Junior ^bc^  Senior ^bc^  Master & PhD ^c^ | 25.0  14.1  12.2  9.5  3.4 | 32.0  33.6  36.1  32.5  25.8 | 26.3  44.8  42.0  47.5  62.9 | 16.7  7.5  9.7  10.5  7.9 | **.001** |
| Student Major Area | Humanities^a^  Soc Sci^b^  Sci & Tech^c^  Cosm & Beauty Sci^d^ | 9.0  15.1  7.2  56.7 | 34.8  40.6  26.4  36.7 | 50.0  37.8  45.0  6.7 | 6.2  6.4  21.4  0.0 | **< .001** |
| Student Political Party | Pan-Blue^ab^  Pan-Green^a^  Environmental^ab^  Others^ab^  Non-affiliated^b^ | 14.2  21.9  0.0  20.0  8.9 | 33.6  32.9  25.0  32.9  32.6 | 38.9  36.8  37.5  40.0  43.2 | 13.3  8.4  37.5  7.1  15.3 | **.003** |
| Univ. Ranking | High^a^  Mid^b^  Low^c^ | 2.1  12.0  32.9 | 14.2  42.0  44.3 | 55.8  43.2  21.1 | 27.9  2.7  1.7 | **< .001** |

Note: Boldface indicates statistical significance (*p* < 0.05). The different superscript letters in the Categories column indicate statistically significant difference between pairs determined by post hoc Dunn tests.

^*^All percentages are calculated on a population of 1118 students.

**S2 Table C. Level of concern about climate change, by demographics^*^**

|  | | | | | | |
| --- | --- | --- | --- | --- | --- | --- |
|  |  | **How concerned are you, if at all, that global climate change will harm you personally at some point in your lifetime? (%)** | | | |  |
|  | **Categories** | **Not at all concerned**  **(%)** | **Not too concerned**  **(%)** | **Somewhat concerned**  **(%)** | **Very**  **concerned**  **(%)** | ***p*-value of**  **Kruskal Wallis** 𝟀^2^ **test** |
| All |  | 1 | 6 | 65 | 28 |  |
| Gender | Male  Female | 1  1 | 7  6 | 65  65 | 27  28 | 0.41 |
| Household Income | < NT$30,000  NT$30,001 - 55,000  NT$55,001 - 80,000  ≥ $80,001 | 3  0  0  1 | 11  4  9  4 | 49  62  66  68 | 37  34  25  27 | 0.15 |
| Student Hometown Region | Center^ab^  East^ab^  North^b^  South^a^ | 1  0  1  0 | 5  0  8  5 | 65  58  67  59 | 30  42  24  36 | **0.0006** |
| Mother’s Education  (Highest *completed*) | Up to Middle School  High School  JR College  BA or Above | 2  1  1  1 | 7  7  4  7 | 61  62  69  64 | 29  30  24  28 | 0.99 |
| Student’s Current Educational Level | Freshmen  Sophomore  Junior  Senior  Master & PhD | 1  0  1  0  1 | 9  4  6  7  7 | 63  70  68  60  56 | 27  26  24  33  36 | 0.23 |
| Student Major Area | Humanities^ab^  Soc Sci^b^  Sci & Tech^a^  Cosm & Beauty Sci^ab^ | 0  1  1  0 | 5  6  8  7 | 70  57  67  77 | 24  34  23  10 | **0.002** |
| Student Political Party | Pan-Blue^ab^  Pan-Green^ab^  Environmental^ab^  Others^a^  Non-affiliated^a^ | 1  1  0  0  0 | 2  5  0  19  7 | 63  60  25  56  66 | 33  33  75  24  25 | **0.003** |
| Univ. Ranking | High  Mid  Low | 1  1  1 | 8  6  7 | 64  66  63 | 27  27  29 | 0.86 |

Note: Boldface indicates statistical significance (*p* < 0.05). The different superscript letters in the Categories column indicate statistically significant difference between pairs determined by post hoc Dunn tests.

^*^All percentages are calculated on a population of 1118 students. They do not add to 100 due to non-responses to some of our questions. “Don’t know” responses were not included; “CC does not exist” and “Not at all Concerned” have been merged into “Not at all concerned”.

**S2 Table D. Degree of behavioral change in response to climate change, by demographics^*^**

|  | | | | | | |
| --- | --- | --- | --- | --- | --- | --- |
|  |  | **How much have you changed your behavior because of climate change? (%)** | | | |  |
|  | **Categories** | **Not at all (%)** | **A little**  **(%)** | **Some**  **(%)** | **Very**  **much**  **(%)** | ***p*-value of**  **Kruskal Wallis** 𝟀^2^ **test** |
| All |  | 3 | 43 | 38 | 11 |  |
| Gender | Male  Female | 4  2 | 42  44 | 34  41 | 13  9 | 0.89 |
| Household Income** | < NT$30,000  NT$30,001 - 55,000  NT$55,001 - 80,000  ≥ NT$80,001 | 7  1  2  2 | 24  41  48  47 | 49  46  37  34 | 18  9  9  11 | **0.04** |
| Student Hometown Region | Centre  East  North  South | 2  0  3  1 | 39  54  45  41 | 44  31  36  41 | 10  11  10  14 | 0.14 |
| Mother’s Education  (Highest *completed*) | Up to Middle School  High School  JR College  BA or Above | 2  3  2  3 | 44  42  45  45 | 43  38  39  37 | 9  12  10  9 | 0.80 |
| Student’s Current Educational Level | Freshmen  Sophomore  Junior  Senior  Master & PhD | 3  2  2  4  3 | 35  48  45  42  45 | 45  34  39  35  38 | 11  11  10  12  7 | 0.09 |
| Student Major Area | Humanities^a^  Soc Sci^b^  Sci & Tech^a^  Cosm & Beauty Sci^ab^ | 2  2  4  0 | 54  37  48  33 | 33  46  35  47 | 10  11  9  13 | **0.0004** |
| Student Political Party | Pan-Blue  Pan-Green  Environmental  Others  Non-affiliated | 2  4  0  3  3 | 45  37  12  49  38 | 36  37  75  29  48 | 11  12  12  16  10 | 0.40 |
| Univ. Ranking | High^a^  Mid^b^  Low^b^ | 2  3  4 | 50  42  36 | 34  38  40 | 7  12  13 | **0.003** |

Note: Boldface indicates statistical significance (*p* < 0.05). The different superscript letters in the Categories column indicate statistically significant difference between pairs determined by post hoc Dunn tests.

^*^All percentages are calculated on a population of 1118 students. They do not add to 100 due to non-responses to some of our questions.

**Pairwise comparisons with post hoc Dunn tests failed to reveal significant differences for household income, despite a significant result for the Kruskal Wallis H test.
