# Supplementary material for: Towards an East Asian model of climate change awareness: A questionnaire study among university students in Taiwan
Source: PLoS One. 2018 Oct 25;13(10):e0206298. doi: 10.1371/journal.pone.0206298 (PMC6201920; doi:10.1371/journal.pone.0206298)
Supplement: S1 Text — (DOCX) [file pone.0206298.s003.docx]

**S1 Text. Climate Change Knowledge Quiz (Chinese)**

**A. 氣候變遷知識與認知**

1、請問氣候與天氣的區別為何？

1.天氣是指在指定的時間與地點的大氣條件。

2.氣候是指發生在一個地點，數年內的平均天氣狀況。

3.氣候變遷，包括濕度，風，但天氣不會。

a. 1

b. 2

c. 3

d. 1與2

2、從1880年開始，哪一個年份是最熱的年份？

a 1970

b 1934

c 1998

d 2015

3、下列哪些不是全球氣候變遷的預測結果？

a 全球更高的氣溫

b 更多的極端天氣

c 導致一些生物的滅絕

d 海平面上升

e 酸雨增加

4、溫室效應發生的原因？

a 溫室氣體發生化學作用而產生額外的熱量

b 地球表面散佈了太多的溫室

c 二氧化碳與其他氣體，讓熱量滯留在大氣中

d 煙囪與火山不斷排放出的硫化物

e由人類活動產生的氣體使更多的熱量離開地球大氣層

5、以下哪一種輻射是可以被溫室氣體所吸收的？

a 可見光

b 紫外線

c 短波輻射

d 紅外線

6、 下列哪個是最有力的的溫室氣體？

a 甲烷

b 二氧化碳

c 臭氧

d 氬氣

7、下列哪一項人類活動造成了最多的人為的二氧化碳排放？

a 燃燒化石燃料

b 森林開墾

c 臭氧層破壞

d 酸沉降

e 農業

8、下列哪一個與氣候變遷有關的溫室氣體成長速度最快？

a 水蒸汽

b 甲烷

c 氟氯碳化合物

d 二氧化碳

e 氧氣

9、下列哪種交通方式，每人每公里的平均消耗能量最多？

a 軌道交通

b 個人汽車（單人出行）

c 公車

d 汽車共乘（司機加乘客，多人出行）

10、太陽能，生物能源，地熱能，水力發電和風能都是“再生能源”。這些被稱為“再生能源”的原因是？

a 不會產生污染

b 都很乾淨

c 很短的時間可以被自然界所補充

d 直接產生電能或熱能

11、 下列哪項作用可以減少大氣中的二氧化碳？

a 光合作用

b 水循環

c 呼吸作用

d 氮循環

12、二氧化碳在大氣中循環一次需要多少時間？

a 1年

b 20年

c 60年

d 100年

13、下列哪個國際條約訂定降低溫室氣體排放的規則？

A 京都議定書

b 蒙特婁(Montreal)協議書

c IPCC協議書

d 上海議定書

14、下列何者正確？

a 氣候變遷可能會減少乾旱頻率

b 更溫暖的氣溫會導致土壤的含水量增加。

c 氣候變遷會增加全球農業產能。

d 海平面上升會淹沒一些世界上農業發達的地區

e 更高的氣溫可以幫助控制農業害蟲

15、從上個世紀以來，地球平均氣溫變化了多少？

a +0.8$℃$

b -0.6℃

c 沒有變化

d +2.0℃

**S1 Text. Climate Change Knowledge Quiz (English)**

1. How are climate and weather different?

1. Weather refers to atmospheric conditions at a specific time and place.

2. Climate refers to the average weather conditions in a place over a period of years.

3. Climate change includes humidity and wind, but weather does not.

a. 1

b. 2

c. 3

d. 1 and 2

2. From 1880 to the present, which of the following years was the hottest?

a. 1970

b. 1934

c. 1998

d. 2015

3. Which of the below is not a predicted impact from global warming?

a. higher temperatures globally

b. more extreme weather

c. extinction of some species

d. rise in mean sea level

e. increased acid rain

4. What factors cause the ‘greenhouse effect’?

a. The chemical reactions of greenhouse gases produce extra heat

b. There are too many greenhouses spread across the surface of the earth

c. Carbon dioxide and other gases retain heat in the atmosphere

d. Sulfides are continuously emitted by chimneys and volcanos

e. Gases produced by human activities cause heat to leave the Earth’s atmosphere

5. Which radiation below could be absorbed by the greenhouse gases in the atmosphere

a. visible light

b. ultraviolet

c. short wave

d. infrared

6. Which of the below is the most potent greenhouse gas?

a. methane

b. carbon dioxide

c. ozone

d. argon

7. Which of these activities releases the most carbon dioxide into the atmosphere?

a. fossil fuel combustion

b deforestation

c ozone loss

d acid rain

e agriculture

8. Which of the gases below associated with climate change has the fastest growth rate?

a water vapor

b methane

c chlorofluorocarbons

d carbon dioxide

e oxygen

9. Which of the following methods of transportation consumes more energy per person per kilometer?

a rail transportation

b personal car (single traveler)

c bus

d car sharing (driver with other passengers)

10. Solar, biomass, geothermal, hydropower and wind energy are all “renewable energy resources”. Why are they called renewable?

a. They don’t produce pollution

b. They are all very clean

c. They can be resupplied by nature in a short time

d. They directly produce electricity or heat

11. Which of the following can reduce atmospheric CO2?

a. photosynthesis

b. the water cycle

c. respiration

d. the nitrogen cycle

12. How long does it take for carbon dioxide to be recycled once in the atmosphere?

a. 1 year

b. 20 years

c. 60 years

d. 100 years

13. Which of the following international treaties sets rules for reducing emissions of greenhouse gases?

a. The Kyoto Protocol

b. The Montreal Protocol

c. The IPCC Agreement

d. The Shanghai Protocol

14. Which of the following is correct?

a. Climate change could reduce the frequency of droughts

b. Warmer temperatures cause an increase in soil moisture

c. Climate change could increase global agricultural production

d. Sea level rise will flood some of the world’s most productive agricultural regions

e. Higher temperatures can help control agricultural pests

15. Since the last century, what has been the change in the Earth’s mean temperature?

a. +0.8°C

b. -0.6°C

c. no change

d. +2.0°C
