# Supplementary material for: Towards an East Asian model of climate change awareness: A questionnaire study among university students in Taiwan
Source: PLoS One. 2018 Oct 25;13(10):e0206298. doi: 10.1371/journal.pone.0206298 (PMC6201920; doi:10.1371/journal.pone.0206298)
Supplement: S2 Text — (DOCX) [file pone.0206298.s004.docx]

**S2 Text. Statistical analysis**

Analyses were performed between July 2016 and November 2017. Three measures of outcome: (1) climate change knowledge; (2) climate change risk perception; and (3) behavioral change served as the dependent variables. Further, the distribution of climate change knowledge scores did not meet the assumption of normality. Given this, and that the other two dependent variables (concern and behavioral change) were ordinal, it was decided to transform the climate change knowledge scores into ordinal categories. The percent correct scores on the 15-item knowledge quiz were transformed to ordinal categories (0-40% = 1; 41-60 = 2; 61-80 = 3; and 81-100 = 4). As the outcome variables were ordinal, we used non-parametric Kruskal-Wallis H tests (one-way ANOVA on ranks) to identify effects of the demographic variables on knowledge, concern and behavioral change. (Results for knowledge as a continuous variable and as an ordinal variable are provided separately; Kruskal-Wallis tests were also used with the continuous knowledge percent scores, as this distribution was non-normal.) When Kruskal-Wallis tests indicated a significant difference, we followed up with a post hoc Dunn test to identify which means among the small subset of all possible pairs differed significantly. Kruskal-Wallis results are provided in Supplementary File S4.

For all three of our dependent variables (knowledge, concern and behavioral change), cumulative odds ordinal logistic regressions with proportional odds were performed to identify significant predictors of the ordinal categories. (Cumulative odds models are used to predict ordinal responses.) Tests for multicollinearity failed to identify any problematic variables. We used goodness of fit tests to assess the extent to which our observed data matched the values predicted by our models. More specifically, we used chi-square goodness of fit (to determine whether the expected and observed frequencies differed significantly) and Nagelkerke pseudo R-square (to compare similar models in terms of how well each is able to explain the observed data) [1]. All analyses were performed with the SPSS statistical package [2].

For climate change knowledge, an ordinal logistic regression was performed to determine the effect of gender, household income, student hometown region, mother’s education, student major, university rank and political party on climate change knowledge. There were proportional odds (i.e., each independent variable was found to have an identical effect at each level of the ordinal dependent variable), as assessed by a full likelihood ratio test comparing the fitted model to a model with varying location parameters (test of parallel lines), χ^2^(4) = 4.916, *p* = 0.296. The percentage of cells with zero frequencies was 10.4%. This model significantly predicted the dependent variable over and above the intercept-only model, χ^2^(2) = 181.053, *p* < .001. University ranking [χ^2^(1) = 136.879, *p* < .001] and family income [χ^2^(1) = 4.082, *p* = .043] both significantly predicted knowledge scores. Odds ratios appear in the main paper, Table 3.

For concern, an ordinal logistic regression was performed to determine the effects of the same set of demographic variables as in the first regression above plus climate change knowledge (percentage correct score) on the likelihood that students would report being more concerned. There were proportional odds (test of parallel lines, χ^2^(20) = 28.533, *p* = 0.097). The percentage of cells with zero frequencies was 50.0%. The overall model was significant (χ^2^(10) = 36.388, *p* < .001). Student hometown region [χ^2^(3) = 12.972, p = .005], political party [χ^2^(4) = 12.250, *p* = .016], and student major [χ^2^(3) = 9.896, *p* = .019], all significantly predicted a higher degree of concern. Odds ratios appear in the main paper, Table 4.

Finally, for behavioral change, an ordinal logistic regression was performed to determine the effects of the same set of demographic variables as in the first regression above plus knowledge and concern on the likelihood of reporting higher levels of behavioral change. There were proportional odds (test of parallel lines, χ^2^(16) = 17.503, *p* = 0.354). The percentage of cells with zero frequencies was 56.6%. The model was significant (χ^2^(8) = 116.265, *p* < .001). Percentage correct score [χ^2^(1) = 9.682, *p* = .002], concern [χ^2^(1) = 81.071, *p* < .001] and student major [χ^2^(3) = 8.981, *p* = .030] were significant predictors of higher levels of reported behavioral change. Student hometown region [χ^2^(3) = 6.963, *p* = .073] was included in the model but was not significant. Odds ratios appear in the main paper, Table 5.

References

1. Bewick V, Cheek L, Ball J. Statistics review 14: Logistic regression. *Critical Care*. 2005 Feb;9(1):112.

2. IBM SPSS Statistics for Windows, Version 20.0. Armonk, NY: IBM Corp; 2011.
